# LASH in Severely Enlarged Uterine Leiomyoma: Removal of a Uterus of 4065 g

**DOI:** 10.1155/2018/2078923

**Published:** 2018-06-25

**Authors:** Garri Tchartchian, Harald Krentel, Bernd Bojahr, Rudy L. De Wilde

**Affiliations:** ^1^Clinic for Minimally Invasive Surgery, Kurstrasse 11, Zehlendorf, 14129 Berlin, Germany; ^2^Department of Obstetrics and Gynecology, St. Anna Hospital, Hospitalstrasse 19, 44649 Herne, Germany; ^3^Clinic for Obstetrics, Gynecology and Gynecologic Oncology, Pius Hospital, University Clinic for Gynecology, Georgstrasse 12, 26121 Oldenburg, Germany

## Abstract

Today, even though minimally invasive approaches have become standard worldwide, large uteri are still mainly removed by means of open abdominal approaches. The present case describes the successful removal of the largest uterus ever reported (4065 g) by means of laparoscopy-assisted supracervical hysterectomy (LASH). We combined LASH with the changeover technique which allows a better access and view. We further explain how this approach allows for the safe minimally invasive removal of uteri of any size.

## 1. Introduction

The continuous development of hysterectomy techniques during the last decades created an apparent shift towards minimally invasive techniques, and the laparoscopic supracervical hysterectomy (LASH) became a routine gynecological procedure. Because of low complication and morbidity rates and reduced risks for the formation of intra-abdominal adhesions or significant scarring, this surgical technique offers patients a safe and mild intervention [1]. Combined with its high level of postoperative satisfaction, short convalescence, short duration of surgery, low postoperative pain level, and, as was recently demonstrated, having a low risk for occult sarcoma (0.06 % [2]), this method evolved into one of the most used laparoscopic hysterectomy approaches worldwide [3]. The present case describes LASH in a patient with a uterus weight of 4065 g, which is, to the best of our knowledge, the largest uterus removed by LASH so far.

## 2. Case Presentation

**Figure 1:**
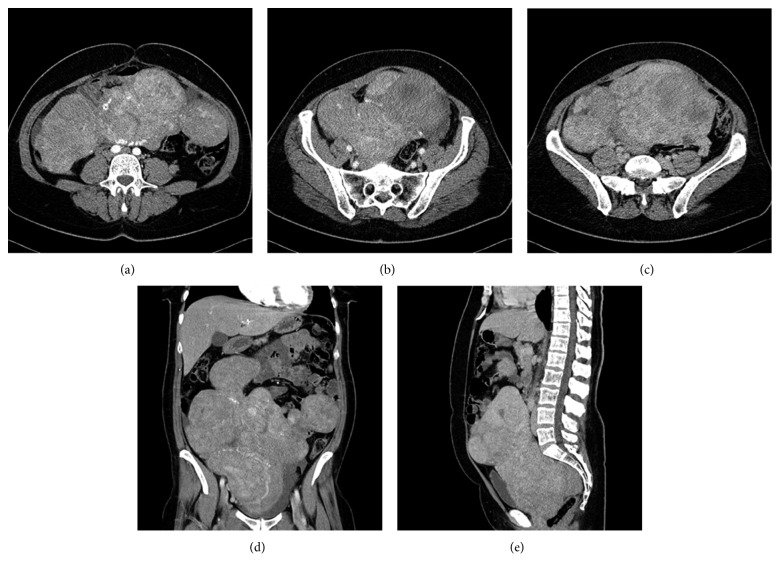
CT scan of the monstrous uterus myomatosus of a 47-year-old nulliparous patient.

A 47-year-old patient with an enormous uterine leiomyoma reaching beyond the navel and up to the costal arch was admitted. During the 14 years since its detection, because of the patient's extreme fear of an abdominal incision, the myoma was merely monitored and all suggested laparotomies thus far had been refused. At the moment of admission, the patient only agreed to a minimally invasive surgery. She was informed in detail about all risks, side effects, and alternatives as well as the potential risk for an emergency open abdominal surgery. Before surgery, we performed imaging diagnostics by means of computed tomography (CT) of the abdomen (Figure 1).

**Figure 2:**
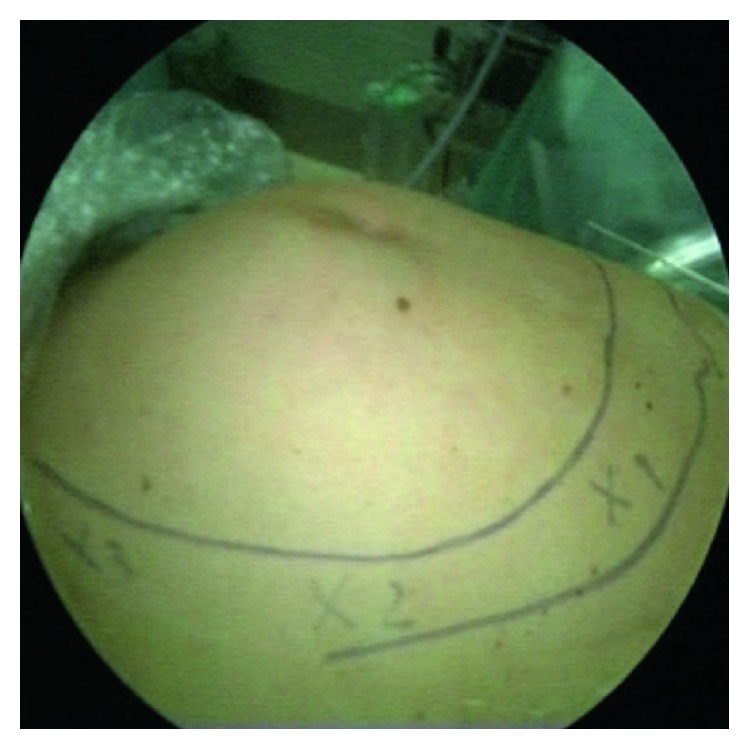
Positions of the 3 trocars on the left side of the patient.

When performing a hysterectomy of a very large uterus (>2500 g), the anatomical changes in the abdomen caused by the size of the uterus need to be taken into account. The large uterus divides the abdominal area, and only 3 narrow spaces are left to manipulate surgical instruments: between the left uterine wall and left abdominal wall, between the right uterine wall and right abdominal wall, and between the fundus uteri and liver and diaphragm. Successful surgery is only feasible when both instruments (forceps and coagulator or scissors) are in the same space simultaneously. Hence, we performed LASH with the “changeover technique” as described previously [4]. As such, we inserted 6 trocars including 3 on the left: one trocar in the lower, one in the middle, and one in the upper abdomen, allowing access to the left narrow space (Figure 2).

**Figure 3:**
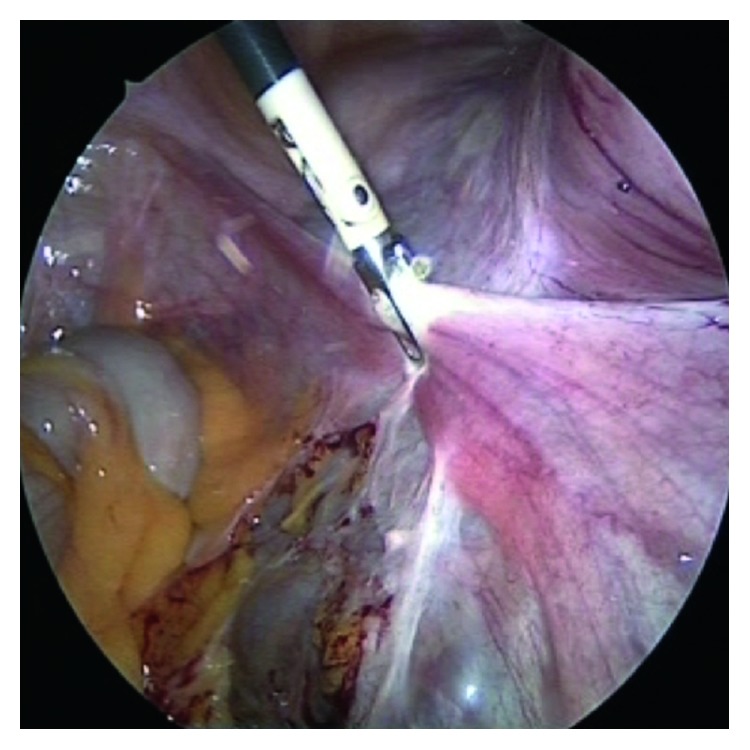
The camera was introduced via the trocar in the upper abdomen.

The 3 trocars on the right side were placed in a mirror-like fashion. Surgery was initiated at the patient's left side, using the upper left trocar to introduce the camera (Figure 3) and the other two left-located trocars to introduce forceps and coagulator or scissors.

The patient was slightly tilted to the opposite side to facilitate visualization and preparation of structures. For uterus manipulation and movement, we used blunt forceps and palpation probes. After parameterization and resection of the uterine adnexa on the patient's left side, the plica vesicouterina was exposed and the bladder was pushed caudally.

**Figure 4:**
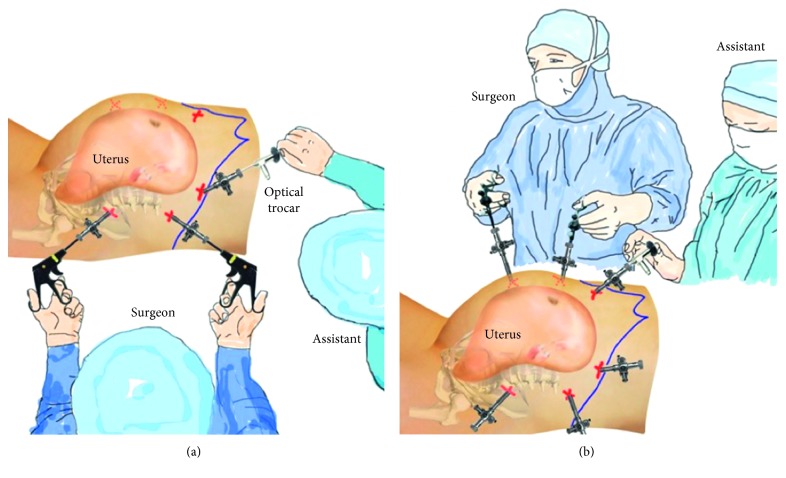
The surgical team switched their position from the left to the right side of the patient which allowed a better access and view of the right side. (a) Start on the left side. (b) Change to the right side.

Next, the surgeon and his team switched sides positioning to the right side of the patient, thus accessing the narrow space on the right side in between the right uterine wall and right abdominal wall (Figure 4). Again, the patient was slightly tilted to the opposite side, enabling better exposure and preparation of organ structures.

**Figure 5:**
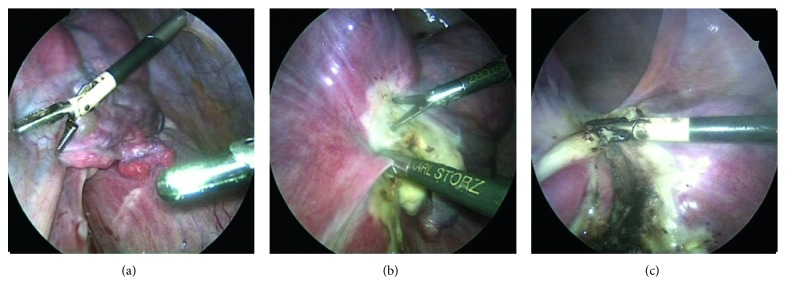
At the patient's right side, the uterus was exposed and the right adnexa, parametria, and blood vessels were prepared and abscised. Then, we resected the corpus uteri and peritonealized the cervix with a purse-string suture (Figure 6).

The procedure continued analogically as on the left side. As was seen on the CT scan, multinodal, intraligamentary, and parametric myomas were found on the right side. Consequently, further exposure and protection of the ureter was done by visualizing followed by preparing or abscising the right adnexa, parametria, and blood vessels (Figure 5).

The corpus uteri were removed from the abdominal cavity by power morcellation. The entire procedure lasted 4 hours and 53 minutes, of which surgery on the uterus took 2 hours and morcellation 2 hours and 53 minutes. No postoperative complications occurred, and the patient was taken to outpatient care 2 days after surgery. Before discharge, a vaginal and renal ultrasound did not reveal any conspicuous intra-abdominal findings. Because of the young age of our patient, a very slow growth of the leiomyoma (over 14 years), and negative Doppler test results before surgery, we assumed no presence of malignancy. Histological analysis of the removed specimen confirmed this assumption. Furthermore, to prevent morcellation-induced spreading of occult malignancy, the patient was alternately tilted from a head-down to head-up position, after which we extensively rinsed the abdominal cavity with Ringer's lactate solution, a routine postsurgical measure at our facility. The total weight of the uterus was 4065 g (Institute for Pathology PPO Berlin; Mona Tawfik, M.D.; 06.05.2014).

## 3. Discussion

Before the development of the changeover technique, it was standard to perform an abdominal hysterectomy to remove very large uteri affected with uterine leiomyoma [4]. Yet, the complication rate of abdominal hysterectomies of large uteri is between 13.1 and 45% [5], while with vaginal hysterectomies, it is merely between 6 and 24% [6]. Still, because of reasons of lack of space, there is a limit in the uterus size which can be removed by means of vaginal hysterectomy. To the best of our knowledge, the heaviest uterus removed by a laparoscopic approach reported thus far was 3030 g [7]. LASH is a method which can surgically remove uteri of almost all sizes [4]. Moreover, our changeover technique enables to complete hysterectomies of uteri of nearly all sizes. The use of the changeover technique is useful and advisable when a high uterine weight of more than 2-2.5 kg is expected. All previously performed surgeries at our clinic with this specific technique were successful and showed no complication (26 uteri > 1500 g). Next to LASH, we also successfully combined the changeover technique with total laparoscopic hysterectomy (TLH) and laparoscopic-assisted combined hysterectomy (LACH) [8]. However, one risk aspect of our method should be mentioned: due to the long duration of morcellation of large uteri, the duration of the entire surgical procedure should not be underestimated. In turn, this increases the risk for thrombosis and neurological complaints [9]. Prophylactic measures to avoid compartment syndrome can reduce this risk significantly [10]. As such, our patient was placed in a supine position with the head resting slightly downwards and not in the lithotomy position.

When combined with the changeover technique, the application of LASH makes the removal of uterus of almost any size feasible. With the changeover technique, it seems possible to complete the dissection of the parametrium at the right side of the uterus without risk. The crucial advantageous aspect of this method is the improvement of visibility and access conditions.

## Figures and Tables

**Figure 6:**
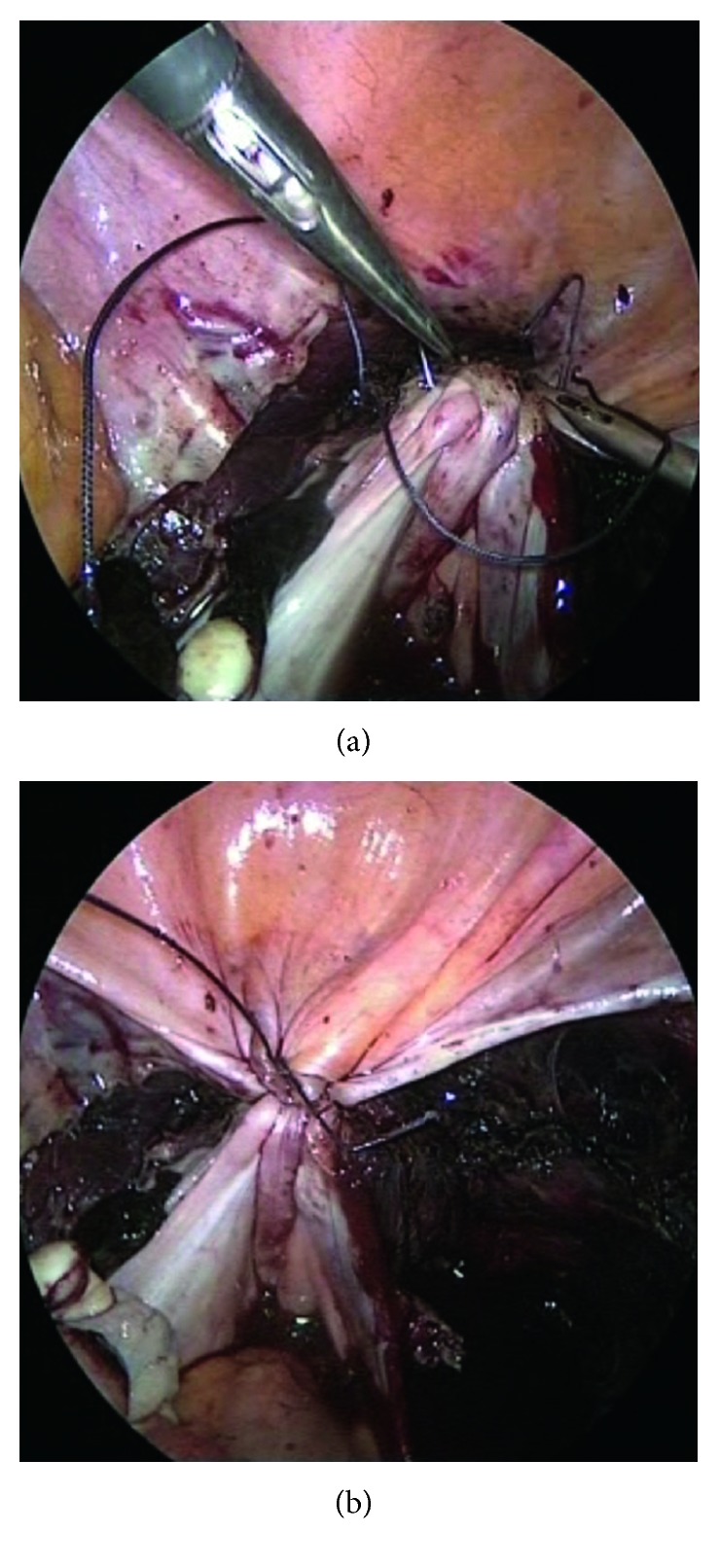
Peritonealization of the cervix with a purse-string suture.
